# Fluctuations of water quality time series in rivers follow superstatistics

**DOI:** 10.1016/j.isci.2021.102881

**Published:** 2021-07-21

**Authors:** Benjamin Schäfer, Catherine M. Heppell, Hefin Rhys, Christian Beck

**Affiliations:** 1 School of Mathematical Sciences, Queen Mary University of London, London E1 4NS, UK; 2 Faculty of Science and Technology, Norwegian University of Life Sciences, 1432 Ås, Norway; 3 School of Geography, Queen Mary University of London, Mile End Road, London E1 4NS, UK; 4 Flow Cytometry Science Technology Platform, The Francis Crick Institute, London, UK

**Keywords:** environmental geochemistry, river geochemistry, statistical physics

## Abstract

Superstatistics is a general method from nonequilibrium statistical physics which has been applied to a variety of complex systems, ranging from hydrodynamic turbulence to traffic delays and air pollution dynamics. Here, we investigate water quality time series (such as dissolved oxygen concentrations and electrical conductivity) as measured in rivers and provide evidence that they exhibit superstatistical behavior. Our main example is time series as recorded in the River Chess in South East England. Specifically, we use seasonal detrending and empirical mode decomposition to separate trends from fluctuations for the measured data. With either detrending method, we observe heavy-tailed fluctuation distributions, which are well described by log-normal superstatistics for dissolved oxygen. Contrarily, we find a double peaked non-standard superstatistics for the electrical conductivity data, which we model using two combined $\chi^{2}$-distributions.

## Highlights

• Fluctuations of detrended water quality time series follow q-Gaussian distributions
• Superstatistical long timescale is extracted from the detrended data
• New type of superstatistics observed, which is well fitted by mixture $\chi^{2}$-distributions

## Graphical abstract

**Figure:**
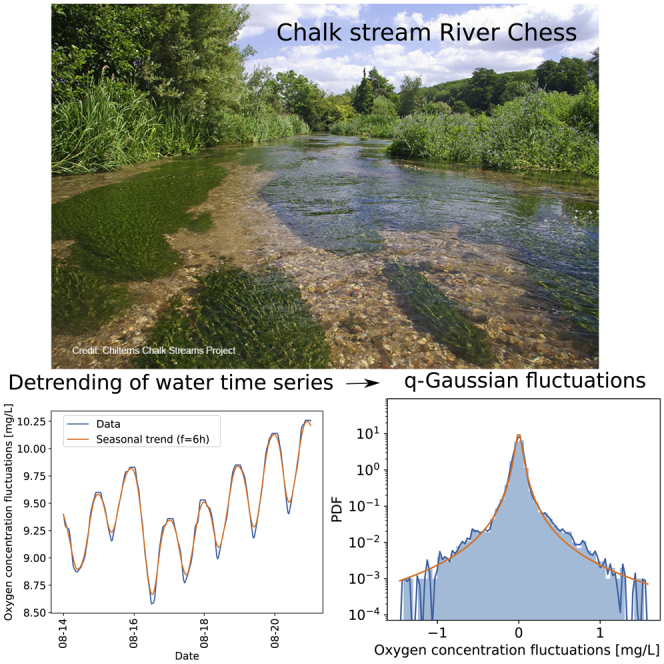

## Introduction

Superstatistical methods, as introduced in (Beck and Cohen, 2003; Beck et al., 2005), provide a general approach to describe the dynamics of complex nonequilibrium systems with well-separated timescales. These models generate heavy-tailed non-Gaussian distributions by a simple mechanism, namely the superposition of simpler distributions whose relevant parameters are random variables, fluctuating on a much larger timescale. Originating in turbulence modeling (Beck, 2007), superstatistics has been applied to many physical systems, such as plasma physics (Livadiotis, 2017; Davis et al., 2019), Ising systems (Cheraghalizadeh et al., 2021), cosmic ray physics (Yalcin and Beck, 2018; Smolla et al., 2020), self-gravitating systems (Ourabah, 2020), solar wind (Livadiotis et al., 2018), high energy scattering processes (Beck, 2009; Sevilla et al., 2019; Ayala et al., 2020), ultracold gases (Rouse and Willitsch, 2017), and non-Gaussian diffusion processes in small complex systems (Chechkin et al., 2017; Itto and Beck, 2021). Furthermore, the framework has successfully been applied to completely different areas, such as modeling the power grid frequency (Schäfer et al., 2018), wind statistics (Weber et al., 2019), air pollution (Williams et al., 2020), bacterial DNA (Bogachev et al., 2017), financial time series (Gidea and Katz, 2018; Uchiyama and Kadoya, 2019), rainfall statistics (De Michele and Avanzi, 2018), or train delays (Briggs and Beck, 2007). The overview article (Metzler, 2020) provides a recent introduction to superstatistics and non-Gaussian diffusion. In all these cases, an underlying simple distribution, typically Gaussian or exponential, is identified to explain the observed heavy tails of the marginal distributions when aggregated with the fluctuating parameter. These tails often decay with a power law. Note that heavy tails are also captured by alpha stable distributions (Shen et al., 2015) or the so-called *κ*-distributions (Livadiotis, 2017). These *κ*-distributions, used in astrophysical plasmas, are a typical example of marginal distributions arising in this context, whereas in statistical physics, one uses the so-called *q*-Gaussians (Tsallis, 2009), with *q* related to *κ* by κ=1/(q−1). Both approaches are equivalent and form standard examples of distributions generated by the (more general) superstatistical approach.

A common feature of real-world time series is that they consist of some long-term trend or oscillation combined with short-term fluctuations. Consider a time series connected to the environment, such as ambient temperature: This will typically display strong seasonal cycles (Kumar et al., 2009). Day-night cycles add another oscillation, while global warming or other long-term influences, such as deforestation, might induce a drift toward higher values. We can decompose the full time series in slower seasonal and drift (trend) terms as well as the short-term fluctuations, using detrending methods. In particular, we consider seasonal detrending, i.e., moving averages, and decomposition via empirical mode decomposition (EMD) (Wu and Huang, 2009), which has recently been shown to disentangle short-term fluctuations from long-term signals (Kampers et al., 2020). Naively, one would expect the so-extracted short-term fluctuations to follow Gaussian distributions.

In this paper, we analyze environmental time series for the River Chess, which is a river located in South East England and is being actively monitored by a citizen science project (Heppell and Treves, 2020). Key questions include how urban areas and a local sewage treatment works affect the water quality. Many different quantities determine the water quality of a river. Here, we focus on two particular quantities: dissolved oxygen concentration and electrical conductivity of the river. Dissolved oxygen (or just “oxygen” for large parts of the paper) is highly relevant for aquatic life, such as fish, in rivers. Meanwhile, electrical conductivity (abbreviated as “EC” or “conductivity”) measures the total dissolved solutes in the water. However, it also measures the impact of humans, e.g., via treated effluent water that is fed into the river. For the current paper, we utilize data available from ChessWatch (Heppell, 2020) and from the four locations Blackwell Hall (BH) [Red], Little Chess (LC) [Blue], Latimer Park (LP) [Green], and Watercress Beds (WB) [Purple]. About twelve months of data collected within the time span of June 2019 to May 2020 are evaluated here. Note that LC and BH are upstream of sewage treatment works, while LP and WB both are downstream of the sewage treatment works. A detailed discussion on how daily cycles influence EC and how machine learning can be used to predict and understand EC trajectories can be found in a future paper (Schäfer et al., 2021). Our main result of the current paper is that the detrended time series behave in a superstatistical way.

This paper is structured as follows. First, we introduce the data and discuss the trajectories and empirical probability density functions (PDFs) of oxygen and EC. Next, we discuss how daily and seasonal cycles are subtracted from the data to reveal the fluctuations. We then continue to present a short recap of superstatistical theory to analyze distributions as generated by a given time series, specifically adapted to our problem here. Finally, we use superstatistical methods to extract long timescales and microscopic distributions of the fluctuating superstatistical parameter *β* as a function of the detrending parameters. Overall, we find that oxygen fluctuations follow approximately log-normal superstatistics, while EC fluctuations point to a new form of superstatistics with a double-peaked *β*-distribution at the LC site.

## Results

### Trajectories and probability distributions

**Figure 1:**
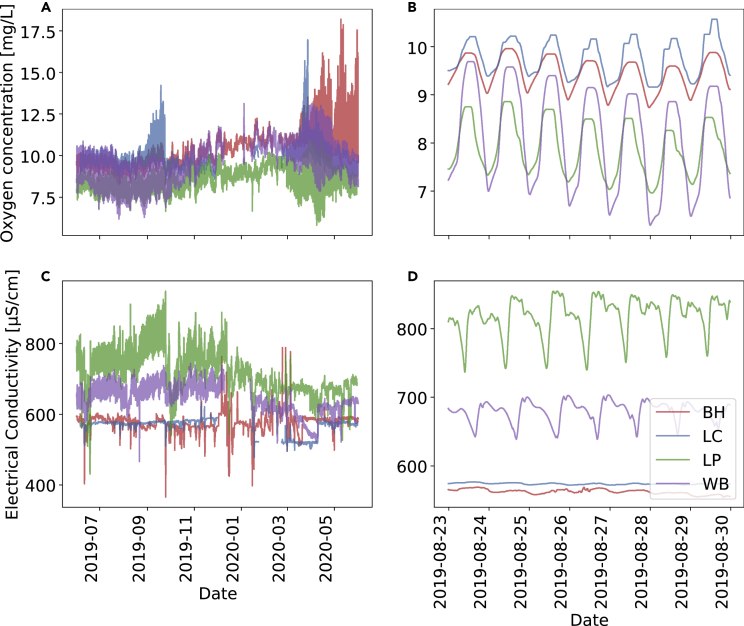
Seasonal and daily cycles Trajectories of the oxygen concentration (A and B) and the electrical conductivity (C and D). We display both the full time period of available data (A and C) and a one-week extract (B and D), highlighting the daily cycles.

To obtain an initial impression of the water quality dynamics, we visualize the trajectories of the oxygen concentration and the electrical conductivity in Figure 1. Disregarding some large peaks at the BH and LC sites, we observe certain seasonal trends in the oxygen trajectories (Figure 1A), i.e., higher concentrations of oxygen in winter to spring than during the summer. On a shorter timescale, both oxygen and electrical conductivity show obvious daily cycles at all stations (Figures 1B and 1D). Electrical conductivity provides a measure of total dissolved solutes in water. Urban streams tend to have higher mean electrical conductivity and major ion concentrations in comparison to their rural counterparts (Conway, 2007; Rose, 2007; Peters, 2009), which arises from a combination of point and diffuse pollution sources. Dissolved oxygen content is a critical indicator of river health for biota, and low dissolved oxygen content or strong daily changes in dissolved oxygen will cause harm to many organisms living in chalk streams such as the River Chess (Arroita et al., 2019; Rajwa-Kuligiewicz et al., 2015).

**Figure 2:**
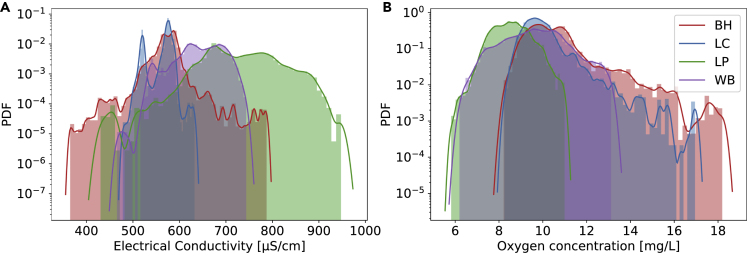
Aggregated statistics points to non-Gaussian dynamics We display the empirical probability density function (PDF) of the electrical conductivity (A) and the oxygen concentration (B). The lines are Gaussian kernel estimates of the empirical PDF. Note the log-scale on the y axis.

Intriguingly, the aggregated statistics shows clear deviations from Gaussianity, see the empirical PDFs of both quantities in Figure 2. In particular, the sites BH and LC (red and blue) display heavy tails. Still, a large portion of the observed variability arises due to daily and seasonal cycles, which we have to subtract from the data before we continue our statistical analysis.

### Detrending

Instead of modeling the full distribution, with its daily and seasonal dynamics, we will describe the fluctuations of the water quality parameters around their respective trend. Detrending reduces the variability and allows for weak stationarity in time series, thus allowing forecasting with more precision (Contreras-Reyes and Idrovo-Aguirre, 2020). To carry out the detrending, we first need to separate the full trajectory *F*(*t*) into trend and fluctuations (assuming an additive model):

(Equation 1) F(t)=Trend(t)+Fluctuations(t).

To achieve this separation, we employ two different methods: seasonal decomposition and EMD.

Seasonal decomposition applies a moving average to the data with a filtering frequency *f* to obtain the trend. The deviation between this moving average and the original data is then classified as fluctuations. Technically, we implement it via the python *statsmodels.tsa.seasonal* package (statsmodel, 2021) and typically apply *f* = 6 hr.

Alternatively, the EMD splits the full trajectory into ordered modes ranging from slowly changing to highly oscillating modes. Similar to a Fourier analysis, summing all modes, it yields the full original data. As has been pointed out recently (Kampers et al., 2020), EMD can be used to disentangle deterministic and stochastic influences. Here, we do the following. All modes *h*_*i*_(*t*) summed up form the full dynamics as follows:

(Equation 2) $$F\left(t\right)=\overset{N}{\underset{i=1}{\sum }}h_{i}\left(t\right),$$

where *N* is the total number of modes. Since the lower numbered modes represent the trend, we keep all but the last *m* modes for the trend and declare the remaining modes as the fluctuations, i.e.

(Equation 3) $$\text{Trend}\left(t\right)=\overset{N-m}{\underset{i=1}{\sum }}h_{i}\left(t\right),$$

(Equation 4) $$\text{Fluctuations}\left(t\right)=\overset{N}{\underset{i=N-m+1}{\sum }}h_{i}\left(t\right).$$

Technically, we implement the EMD via the PyEMD package (Laszuk, 2017) and chose *m* = 2 for most cases.

**Figure 3:**
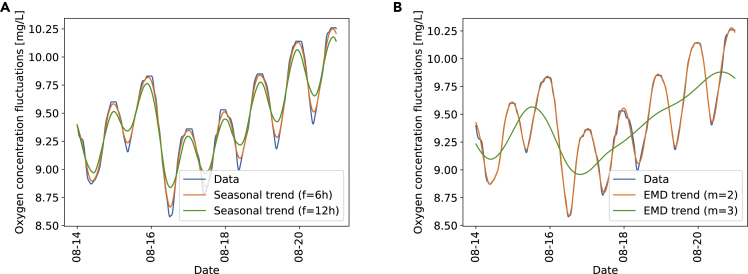
Illustration of the data detrending We apply seasonal detrending (A) or detrending via EMD (B). The data (blue) are best approximated by a filtering frequency of *f* = 6*h* and dropping *m* = 2 modes, respectively (orange). Choosing a larger *f* or *m* oversimplifies the dynamics (green), while smaller settings would overfit the noise. Here, we plot a one-week extract of the oxygen trajectory for the BH measurement site. Note that the EMD is still carried out on the full dataset, as the number of modes per individual week would vary otherwise.

Both detrending procedures are demonstrated in Figure 3 using oxygen concentrations from the BH measurement site. The orange curves, corresponding to a filtering frequency of *f* = 6*h* or dropping *m* = 2 modes, describes the trend of the data well, while preserving short timescale fluctuations. These parameter settings are a compromise between barely capturing any trend (green curves) and overfitting (essentially reproducing the blue data). We will later study the effect of the detrending parameters on the superstatistical results systematically. With the data separated into trend and fluctuations, let us now continue to investigate the fluctuation statistics using a superstatistical approach.

### Superstatistical time series analysis

The basic idea of superstatistics (Beck and Cohen, 2003; Beck et al., 2005) is the concept that a longer time series with a complicated and often heavy-tailed probability distribution is indeed an aggregation of many shorter time series, each giving rise to a simple, non-heavy-tailed distribution. Superstatistical methods have been successfully applied to many different types of complex systems (Beck and Cohen, 2003)–(Metzler, 2020). As a first step of superstatistical time series analysis, we will have to extract a long timescale *T* on which we locally observe simple distributions. Assume we know that locally, in shorter time slices, the time series is approximately Gaussian distributed. In this case, the kurtosis of a local snapshot should be $\kappa_{\text{Gaussian}}=3$. In contrast, the fully aggregated time series will display a much higher kurtosis *κ*. To determine *T*, we test different time window sizes Δt and compute the local average kurtosis (Beck et al., 2005) as follows:

(Equation 5) $$\bar{\kappa }\left(\text{Δ}t\right)=\frac{1}{t_{max}-\text{Δ}t}\int_{0}^{t_{max}-\text{Δ}t}dt_{0}\frac{\langle {\left(u-\bar{u}\right)}^{4}\rangle_{t_{0},\text{Δ}t}}{\langle {\left(u-\bar{u}\right)}^{2}\rangle_{t_{0},\text{Δ}t}^{2}},$$

where *t*_*max*_ is the length of the time series and $\langle \ldots \rangle_{t_{0},\text{Δ}t}$ is the expectation for the time slice of length Δt starting at *t*_0_. The long timescale is then assumed as $\bar{\kappa }\left(T\right)=\kappa_{\text{Gaussian}}$, i.e., the average kurtosis of windows of length *T* has a Gaussian kurtosis $\bar{\kappa }\left(T\right)=3$. After determining *T*, we can split the time series in several samples, each of length *T* and thereby obtain a collection of approximately local Gaussian distributions, each with a different inverse variance parameter *β*. If these *β* themselves follow a $\chi^{2}$-distribution, then it can be written as follows:

(Equation 6) $$f\left(\beta \right)=\frac{1}{\text{Γ}\left(\frac{n}{2}\right)}{\left(\frac{n}{2\beta }\right)}^{\frac{n}{2}}\beta^{\frac{n}{2}-1}e^{-\frac{n\beta }{2\beta_{0}}},$$

with *n* being the degrees of freedom for the distribution and $\beta_{0}$ the mean of *β*; we then analytically obtain a *q*-Gaussian for the aggregated statistics (Beck, 2001; Beck et al., 2005). Alternatively, the *β*-distribution might be well described by some other distribution, such as an inverse $\chi^{2}$ or log-normal distribution. In this case, the marginal distribution obtained by integrating over *β* is different (though often, in good approximation, well approximated by a *q*-Gaussian). A given time series is then said to follow $\chi^{2}$, inverse $\chi^{2}$, or log-normal superstatistics, depending on what the actual distribution of *β* is. As superstatistics was originally derived for temperature fluctuations, *β* is often interpreted as an inverse temperature (Uchiyama and Kadoya, 2019), related to the local kinetic energy in the system. But in general, it is just a fluctuating inverse variance parameter of a given time series.

For generic superstatistics, we expect to observe in good approximation *q*-Gaussian PDFs, which are given as follows:

(Equation 7) $$p\left(q,b,\mu \right)=\frac{\sqrt{b}}{C_{q}}{\left(1+\left(1-q\right)\left(-b{\left(x-\mu \right)}^{2}\right)\right)}^{\frac{1}{1-q}},$$

where *C*_*q*_ is the normalization constant, *μ* is a shift parameter, *q* is a shape parameter, also known as the entropic index, and *b* is a scale parameter proportional to the expectation ⟨β⟩ as formed with the distribution given in Equation (6). For *q*→1, *q*-Gaussians become Gaussian distributions with variance 1/2*b*. For a specialized book on the applications of *q*-statistics in water engineering, see (Singh, 2016). Note that the superstatistical distributions described here may arise from a Gaussian process if such a process has a time-dependent standard deviation, i.e., displays a superposition of simple Gaussian distributions, in the long term.

While the long timescale *T* describes the timescale on which the underlying stochastic process changes, the short timescale *τ* gives the time for the system to relax toward its (local) equilibrium. It is defined by evaluating the decaying autocorrelation of the original time series, approximated by $c∼{\text{e}}^{\left(-t/\tau \right)}$. To ensure that the system can always relax to its new equilibrium, we have to assume *τ*≪*T* for the superstatistical approach to hold. We validate this in the supplemental information.

### Superstatistical analysis for the river chess

**Figure 4:**
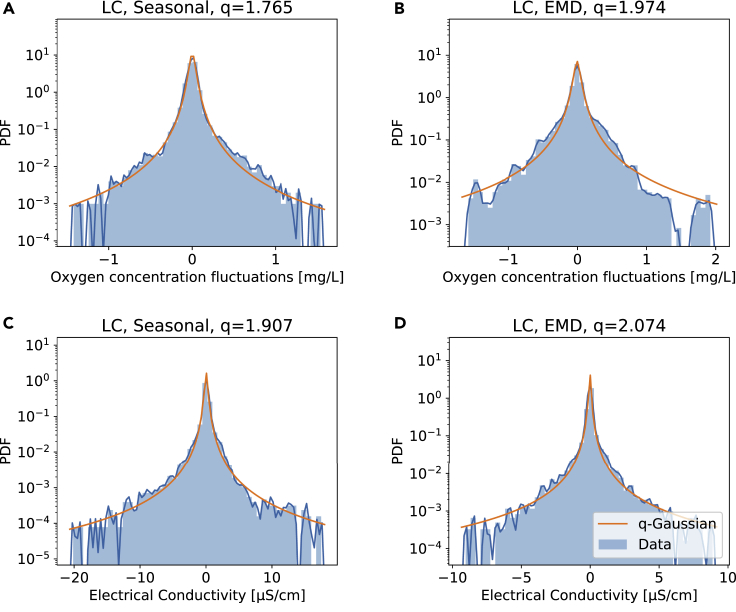
Detrending of the data reveals non-Gaussian fluctuations, approximated by *q*-Gaussians in both cases We plot the empirical probability density functions (PDFs) of detrended oxygen concentrations (A and B) and electrical conductivity (C and D). Regardless whether the detrending is carried out via seasonal detrending (A and C) or EMD (B and D) leads to these non-Gaussian distributions, which are well approximated by *q*-Gaussian distributions. The blue lines are Gaussian kernel estimates of the empirical PDF. The orange fits of *q*-Gaussians were obtained via maximum likelihood estimation (MLE), see code for details.

With the data detrended and the superstatistical foundations laid out, let us investigate the fluctuations in the two time series for the River Chess data (oxygen and electrical conductivity). First, we note that the detrending of either water quality parameter leaves us with a highly non-Gaussian distribution, which is well captured by a *q*-Gaussian distribution, see Figure 4.

**Figure 5:**
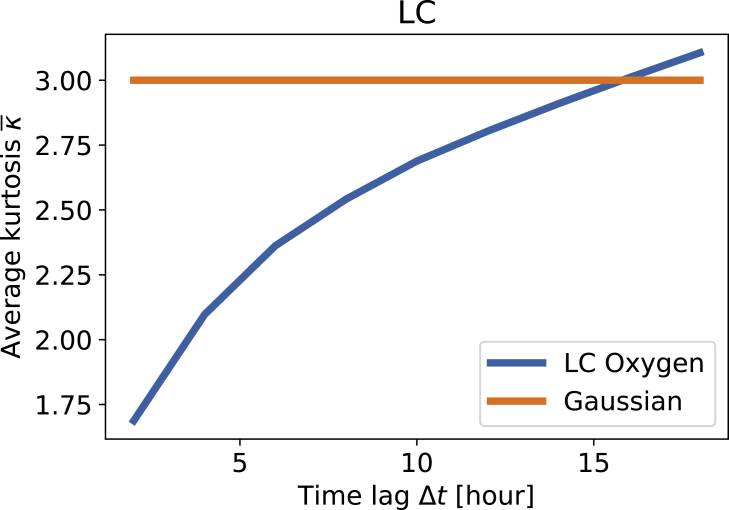
The long timescale is determined using the average kurtosis Specifically, we display the average kurtosis $\bar{\kappa }$ as a function of the time window Δt and determine *T* from the condition κ(T)=3. Assuming Gaussian distributions locally in a window of length *T*, they have kurtosis 3, whatever their variance. In this way, for the LC site displayed here, we obtain *T* ≈ 16*hr*.

To investigate how these non-Gaussian distributions could arise, we continue with the superstatistical ansatz: Let us assume that the non-Gaussian fluctuations arise from local Gaussian distributions. If this was the case, we could extract a long timescale *T* on which the distribution is locally a Gaussian distribution. We determine this long scale as the time window for which the average kurtosis $\bar{\kappa }$ of the data is $\bar{\kappa }\left(T\right)=\kappa_{\text{Gauss}}=3$, see Figure 5. For the LC measurement site, using seasonal detrending and investigating oxygen concentrations, we observe a long timescale of *T*_LC_≈16 *hr*.

**Figure 6:**
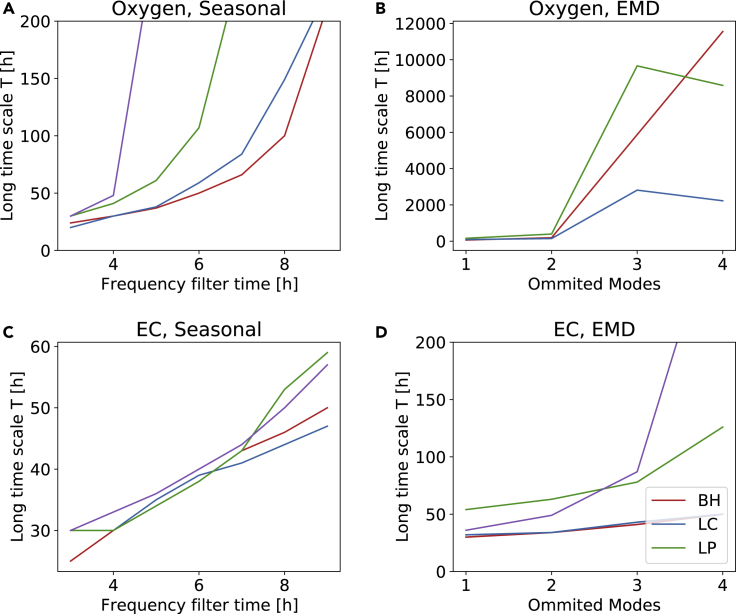
Long timescales *T* scale almost linearly with the detrending parameters before the description breaks down We plot the obtained long timescale *T* for the detrended oxygen concentrations (A and B) and electrical conductivity (C and D), considering both detrending via seasonal detrending (A and C) or EMD (B and D). If the number of omitted modes *m* or the filtering frequency *f* is set too high, the average kurtosis always remains below $\kappa^{\text{Gaussian}}=3$, and hence, no timescale *T* is determined in this case.

Let us continue this investigation more systematically. Namely, as pointed out above, the detrending method and detrending parameter (filtering frequency *f* and number of omitted modes *m*) will likely influence the superstatistics and thereby the long timescale. Hence, we visualize this dependency for both methods and both quantities in Figure 6. Apparently, the long timescale scales approximately linearly with the detrending parameter in a certain parameter range. Then, when the detrending parameter is increased too much (e.g. at *f* > 4*h* for seasonal detrending and oxygen or *m* > 2 for EMD and oxygen), the long timescale suddenly increases dramatically. This can be explained as follows: The influence of the specific detrending parameter on the long timescale is moderate as long as the derived fluctuation distribution is heavy tailed. If too many modes are attributed to the fluctuations (large *m*) or too high frequencies are used (high filter frequency *f*), then the fluctuation distributions might only barely be heavy tailed (high *T*) or display a platykurtic behavior, i.e., a kurtosis *κ* < 3. Based on the results seen here, we are confident that a filtering frequency of *f* = 6*h* and attributing *m* = 2 modes to the fluctuations yields solid results for as many cases as possible. The special case of the WB site, which would require *f* ≤ 4*h* is thereby not included to avoid overfitting at the other sites. With the method established, let us carry out two consistency checks: snapshots and *β*-distribution.

**Figure 7:**
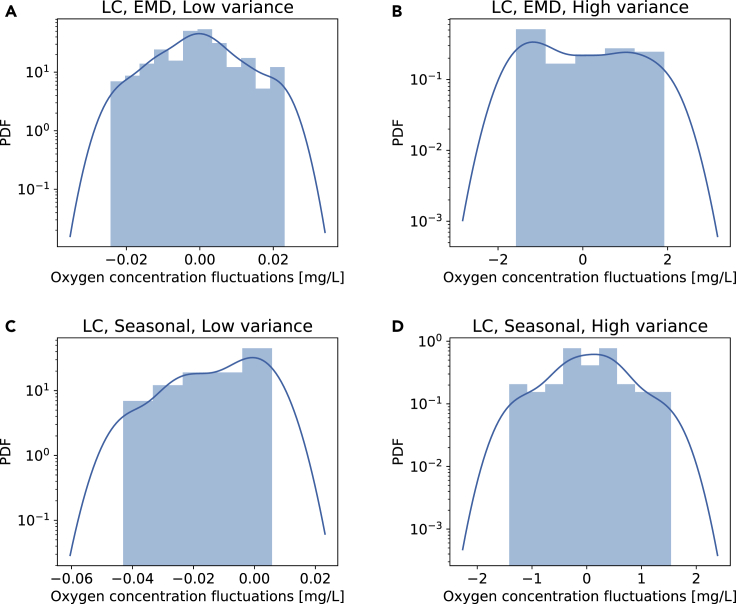
Local snapshots of length *T* are approximated by Gaussian distributions We consider both EMD detrending (A and B) and seasonal detrending (C and D) and display for both cases a window of length *T*, selecting cases with lowest variance (A and C) and the highest variance (B and D). All plots are for the LC site data. The figure illustrates how strongly the local variance fluctuates. The blue lines are Gaussian kernel estimates of the empirical PDF.

First, we inspect snapshots of the fluctuation trajectory of length *T*. According to the superstatistical approach, these local snapshots should follow a Gaussian distribution. Indeed, inspecting the plots in Figure 7, we observe approximately Gaussian distributions. Note that the long timescale here is of the order of 10-100 hr and the data have 15 min resolution, i.e., each local snapshot contains ~ 100-1000 measurements.

**Figure 8:**
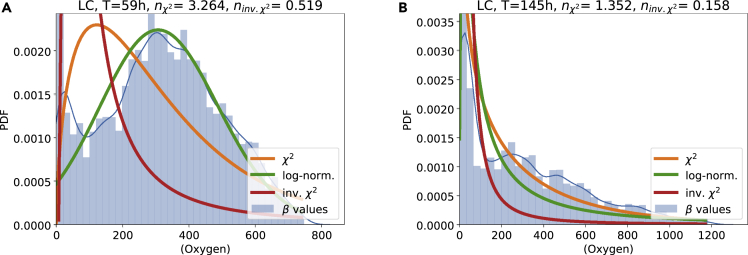
The extracted *β*-distribution of the oxygen concentration fluctuations is well approximated by a log-normal fit Here, we assume local Gaussian distributions (with fluctuating variance in each time slice) and investigated the LC measurement site, considering both seasonal detrending (A) and EMD (B). The blue lines are Gaussian kernel estimates of the empirical PDF.

**Figure 9:**
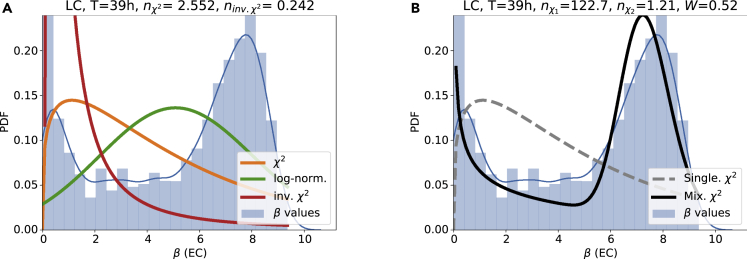
The extracted *β*-distribution of the electrical conductivity fluctuations does follow a mixture of two $\chi^{2}$-distributions (A) *β*-distribution with $\chi^{2}$, inverse $\chi^{2}$, and log-normal fit. (B) *β*-distribution with a single and the mixture $\chi^{2}$-distribution fitted. Both plots use seasonal detrending at the LC measurement site. The blue lines are Gaussian kernel estimates of the empirical PDF.

Finally, we compute the distribution of the effective damping to noise ratio *β*. The superstatistical hypothesis implies that the observed heavy tails (fitted *q*-Gaussian-like distributions in Figure 4) arise either exactly from $\chi^{2}$ or approximately from inverse $\chi^{2}$ or log-normal distributions of *β*. Here, we observe something very interesting: While the *β*-distributions of the oxygen fluctuations are well approximated via log-normal or alternatively $\chi^{2}$ distributions (Figure 8), the *β*-distributions of the electrical conductivity fluctuations do follow a very different type of distribution (Figure 9). While the *β*-distribution for oxygen is single-peaked, the one of the electrical conductivity displays two peaks: One close to zero and one at larger values of *β*. These distributions with two peaks are somewhat unusual distributions, typically not encountered in the standard superstatistics formalism. They provide something new and are specific to the data analyzed here. Remember that the electric conductivity is heavily influenced by human influences, such as the outflow of the sewage treatment works, which could be the deeper reason for the observed unusual behavior: The single-peaked *β*-distributions at the LP and BW sites could arise due to human influence, while the double-peaked *β* distributions at the LC site might hint at complex natural processes, e.g., interaction of rainfall events or the flora and fauna with the conductivity fluctuations.

#### Mixture of $\chi^{2}$-distributions

Let us search for a suitable description of the double-peaked *β*-distribution observed for electrical conductivity. As a simple extension of a single $\chi^{2}$-distribution, we propose to use a mixture distribution of two $\chi^{2}$-distributions:

(Equation 8) $$f\left(\beta \right)=Wf_{\chi^{2}}\left(\beta ,n_{\chi_{1}},\beta_{0}\right)+\left(1-W\right)f_{\chi^{2}}\left(\beta ,n_{\chi_{2}},\beta_{0}\right),$$

i.e., the full *β*-distribution is composed as a sum of two $\chi^{2}$-distributions, sharing a $\beta_{0}$ parameter (originally the mean β) and each having its own degree of freedom $n_{\chi_{1}}$ and $n_{\chi_{2}}$. Both distributions are weighted by the weight constant *W*, which ranges from 0 to 1.

Indeed, this new mixture distribution of two $\chi^{2}$-distributions is an excellent fit to the data, see Figure 9 and Supplements for further examples.

## Discussion

In this paper, we have shown that environmental time series relevant for water quality in chalk rivers, such as the River Chess, behave in a superstatistical way. We observe heavy-tailed distributions for the aggregated statistics of oxygen and electrical conductivity. The dynamics of the measured time series is consistent with that of a nonstationary process consisting of patches that locally exhibit Gaussian behavior, with the variance parameter fluctuating on a longer timescale *T*, which we extracted from the data. A new result is that the fluctuations of these water quality parameters do not follow Gaussian distributions as a whole but have distinct heavy tails that are well approximated by *q*-Gaussian functions. This result is observed regardless of which detrending method (seasonal detrending and EMD) is applied. Using the average kurtosis, we determined the long timescale *T* and found that the detrending method and specific detrending parameter only lead to linear scaling of the deduced long timescale, i.e., the superstatistical finding as such is robust with respect to the specific detrending method. Consistent with the superstatistical assumptions, the local snapshots follow approximately Gaussian distributions, and the *β*-distribution of oxygen fluctuations are approximated by log-normal distributions, quite a similar statistics as the one known for velocity and acceleration fluctuations in hydrodynamic turbulence.

An intriguing new finding is that electrical conductivity fluctuations at the LC site (contrary to oxygen fluctuations) display an unusual statistics, namely a double-peaked *β*-distribution that is not immediately captured by existing superstatistical theory. We demonstrated how a $\chi^{2}$ mixture distribution can approximate the results, but still, this finding points to the need of additional theoretical models that lead to double-peaked *β*-distributions. As a first step toward this extended theory, we propose a mixture $\chi^{2}$. Other possibilities to extend superstatistics could include bivariate superstatistics (Caamaño-Carrillo et al., 2020).

Our superstatistical analysis requires the initial detrending of the data, illustrating that fluctuations of environmental time series are generally not homogeneous in time. The data analyzed here are somewhat comparable to other environmental time series with seasonal influence, e.g., the analysis of ambient temperature (Yalcin and Beck, 2013). Our approach could be applied to other seasonal time series: First, decompose the full time series into trend and fluctuations and then extract the distributions of the fluctuations as being heavy tailed, followed by further superstatistical analysis to extract the relevant timescales and distributions of the parameter *β*.

Interestingly, the impact of the sewage treatment works on the heavy tail statistics is limited: Regardless of location, we did observe similar highly non-Gaussian distributions of the fluctuations, i.e., both upstream and downstream of the sewage treatment site (Figure 4). Contrary, the long timescale, especially when using seasonal detrending on oxygen and EMD on electrical conductivity, displays qualitatively different behavior for the upstream and downstream locations (Figure 6), illustrating that human influence can be seen via timescale parameters extracted from the superstatistical analysis. In particular, the long timescales diverge for lower filtering parameters at the two downstream locations with lower oxygen and higher electrical conductivity values. Meanwhile, we observe that the doubled-peaked *β*-distribution is most pronounced for the LC and BH sites (upstream). This might indicate that the double peaked *β*-distribution can emerge due to natural fluctuations, while fluctuations at the two downstream locations (LP and WB) might be further influenced by the human activity. Still, further research is necessary to fully understand this aspect. Finally, we note that the observed *q*-Gaussians imply a larger number of extreme events compared to any Gaussian process and the presented superstatistical approach provides a means to quantify this.

A future project would be to compare our results obtained for the River Chess with the statistics generated by other environmental time series, in particular comparing different rivers in a systematic and quantitative way or include other parameters (Kumar, 2011). Moreover, from a theoretical point of view, it would be desirable to expand the superstatistical theory relevant in nonequilibrium statistical physics toward double-peaked *β*-distributions, as these distributions seem to appear naturally in the environmental context.

## STAR★Methods

### Key resource table

**Table:** 

REAGENT or RESOURCE	SOURCE	IDENTIFIER
**Software**		

Python3	https://www.python.org	
Numpy	https://numpy.org	version 1.20.0
Scipy	https://www.scipy.org	version 1.6.0
Seaborn	https://seaborn.pydata.org/	version 0.11.1
PyEMD	https://pypi.org/project/EMD-signal/	version 0.2.15

### Resource availability

#### Lead contact

Benjamin Schäfer (benjamin.schaefer@nmbu.no)

#### Materials availability

This study did not generate new unique reagents.

#### Data and code availability

All code to reproduce the results presented here along the necessary data (both in original and in cleaned and detrened form) are available at: https://osf.io/mxcrv/

### Method details

All calculations included in this manuscript were performed using Python and the libraries referenced above. All information necessary to reproduce these results are included in the main body of the text and in the OSF repository.

## Footnotes

Supplemental information can be found online at https://doi.org/10.1016/j.isci.2021.102881.
