# Supplementary material for: Fluctuations of water quality time series in rivers follow superstatistics
Source: iScience. 2021 Jul 21;24(8):102881. doi: 10.1016/j.isci.2021.102881 (PMC8348929; doi:10.1016/j.isci.2021.102881)
Supplement: Document S1. Figures S1–S3 [file mmc1.pdf]

iScience, Volume *24*

Supplemental information

Fluctuations of water quality time series in rivers follow superstatistics

Benjamin Schäfer, Catherine M. Heppell, Hefin Rhys, and Christian Beck

Supplementary Information for
Fluctuations of water quality time series in rivers follow superstatistics

Benjamin Schäfer,[1,2,*] Catherine M. Heppell,[3] Hefin Rhys,[4] and Christian Beck[1]

[1] *School of Mathematical Sciences, Queen Mary University of London, London E1 4NS, United Kingdom*
[2] *Faculty of Science and Technology, Norwegian University of Life Sciences, 1432 Ås, Norway*
[3] *Queen Mary University of London, School of Geography, Mile End Road, London E1 4NS, UK*
[4] *Flow Cytometry Science Technology Platform, The Francis Crick Institute, London, UK*

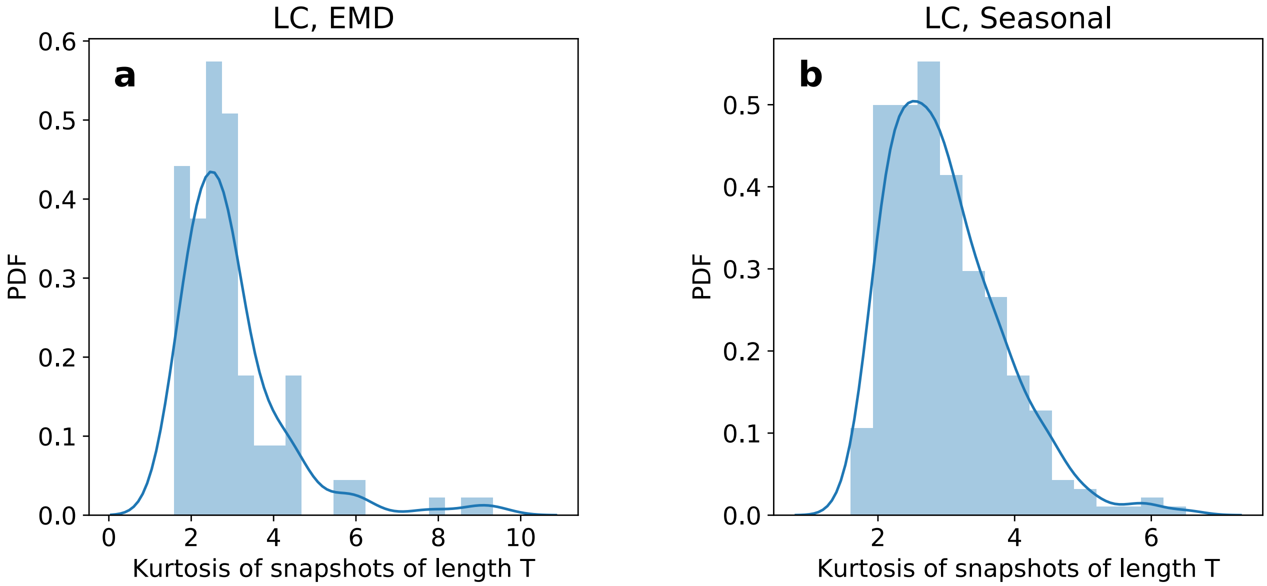

FIG. 1. Kurtosis distribution of trajectory snapshots of length T. On average, we observe a Gaussian distribution (see also Fig. 5 in the main text) but locally each distribution might be slightly leptokurtic or platykurtic, i.e. have a kurtosis larger or smaller than 3, see also STAR Methods.

* Correspondence: benjamin.schaefer@nmbu.no

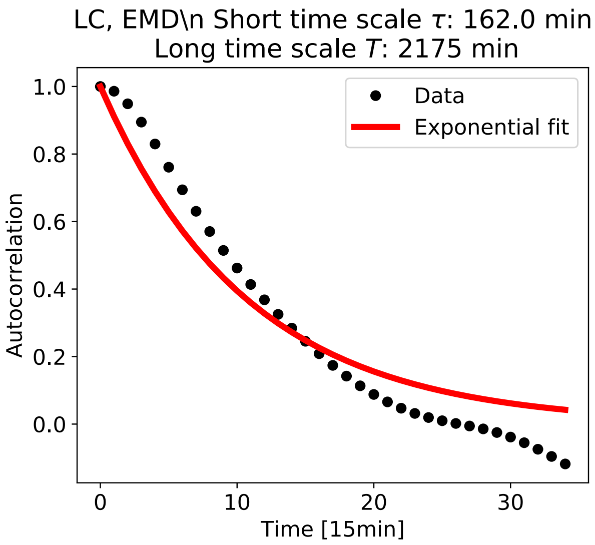

FIG. 2. Time separation: The short time scale τ, as determined by the approximately exponential autocorrelation decay $c(t) \sim \mathrm{e}^{(-t/\tau)}$ is at least one order of magnitude faster than the long time scale T, as determined in Figures 5 and 6 of the main text. Here, we show this for the LC site, using EMD detrending when analysing dissolved oxygen, see also STAR Methods.

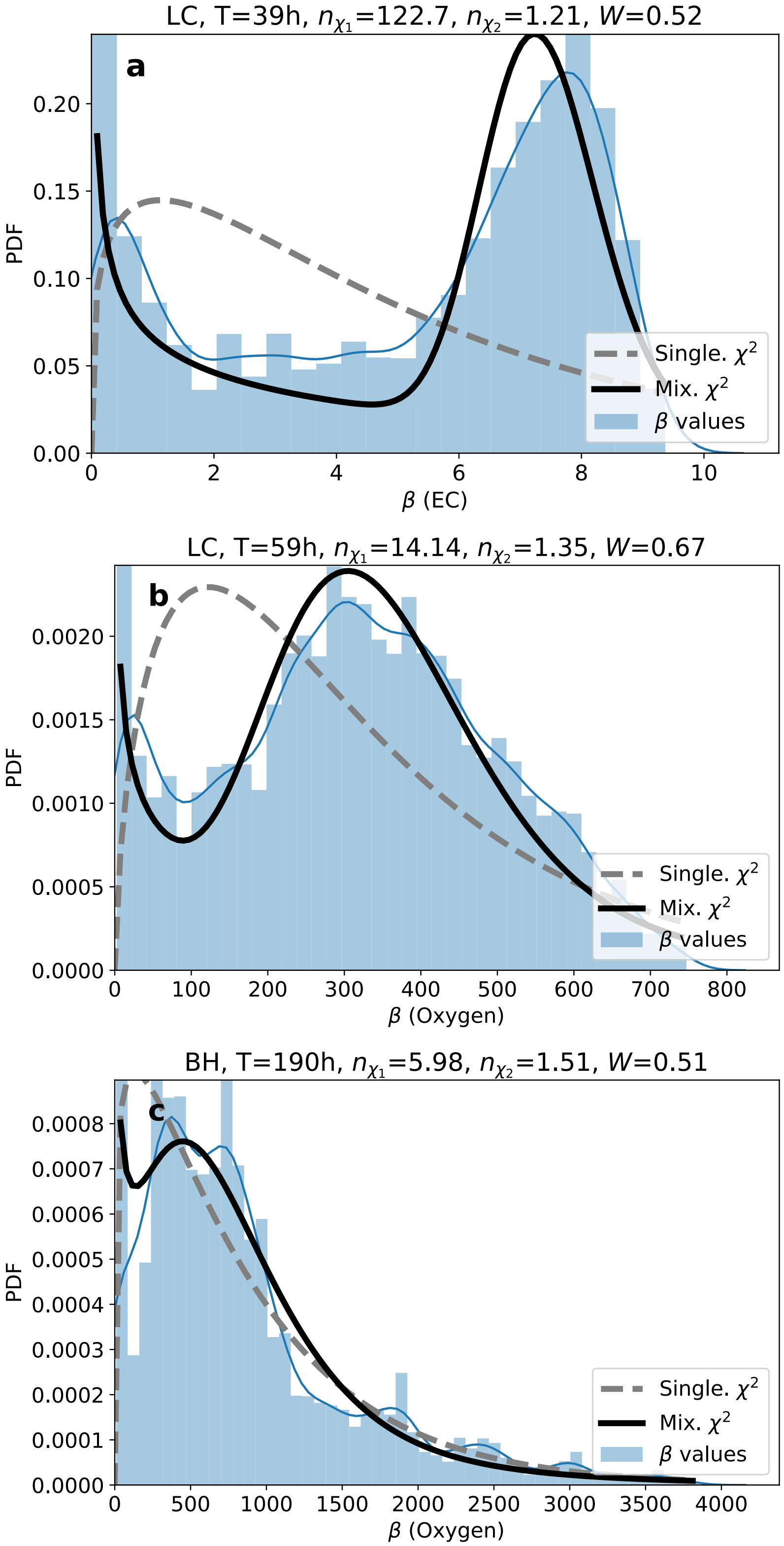

FIG. 3. Examples of mixture χ^2 distributions, complementing Figure 9 from the main text. a: Sesonal detrending of EC measurements at the LC site, b: Sesonal detrending of oxygen measurements at the LC site, c: EMD detrending of oxygen measurements at the BH site, see also STAR Methods. The mixture β distribution is given by $f(\beta) = W f_{\chi^2}(\beta, n_{\chi_1}, \beta_0) + (1 - W) f_{\chi^2}(\beta, n_{\chi_2}, \beta_0)$.
